# Antibiotic-resistant *Campylobacter coli* and *Campylobacter jejuni* in commercial and smallholder farm animals in the Asante Akim North Municipality of Ghana

**DOI:** 10.3389/fmicb.2022.983047

**Published:** 2022-11-04

**Authors:** Ellis Kobina Paintsil, Linda Aurelia Ofori, Charity Wiafe Akenten, Andreas E. Zautner, Joyce Mbwana, Anna Jaeger, Maike Lamshöft, Jürgen May, Kwasi Obiri-Danso, Richard Odame Philipps, Ralf Krumkamp, Denise Dekker

**Affiliations:** ^1^Kumasi Center for Collaborative Research in Tropical Medicine (KCCR), Kumasi, Ghana; ^2^Department of Theoretical and Applied Biology, Kwame Nkrumah University of Science and Technology, Kumasi, Ghana; ^3^Institute of Medical Microbiology and Hospital Hygiene, Medical Faculty, Otto-von-Guericke University Magdeburg, Magdeburg, Germany; ^4^National Institute for Medical Research (NIMR), Tanga, Tanzania; ^5^Bernhard Nocht Institute for Tropical Medicine (BNITM), Hamburg, Germany; ^6^German Center for Infection Research (DZIF), Partner Site Hamburg-Lübeck-Borstel-Riems, Hamburg, Germany; ^7^Tropical Medicine II, University Medical Center Hamburg-Eppendorf (UKE), Hamburg, Germany

**Keywords:** *Campylobacter coli*, *Campylobacter jejuni*, commercial farms, smallholder farms, antimicrobial resistance, Ghana

## Abstract

Worldwide, farm animals, in particular poultry, are an important reservoir for *Campylobacter* spp. However, information on *Campylobacter* colonization in farm animals in Africa is scarce. Hence, this cross-sectional study determined antibiotic-resistant *Campylobacter* from both commercial and smallholder farm animals in the Asante Akim North Municipality of Ghana. Fecal samples from poultry and livestock kept by commercial and smallholder farms were collected and analyzed using standard microbiological methods. The overall *Campylobacter* frequency was 20.3% (*n*/*N* = 322/1,585), and frequencies detected were similarly high in isolates from commercial (21.0%, *n*/*N* = 169/805) and smallholder (19.6%, *n*/*N* = 153/780) farms. Species isolated were *C*. *coli* (67.7%, *n*/*N* = 218/322) and *C*. *jejuni* (32.3%, *n*/*N* = 104/322). However, the frequency of *C*. *coli* was 2.1 (95% CI: 1.8–2.5) times higher than what was found for *C*. *jejuni*. *Campylobacter* frequencies in the rainy season was 22.2% (*n*/*N* = 258/1,160) and 15.1% (*n*/*N* = 64/425) in the dry season (prevalence ratio = 1.48, 95% CI: 1.2–1.9). About 1.7% (*n*/*N* = 6/322) of the *Campylobacter* isolates, all from smallholder farms, were susceptible to all antibiotics tested. Multidrug resistance was observed for 4.7% (*n*/*N* = 15/322) of the *Campylobacter* isolates, of which 93.3% (*n*/*N* = 14/15) occurred in isolates from commercial farms. This study highlights the need for the implementation of control programs, in commercial farming but also at the smallholder farm level, to formulate clear guidelines aimed at decreasing *Campylobacter* contamination of meat products and reducing the use of antibiotics in the farming sector.

## Introduction

*Campylobacter* spp. are ubiquitous in the farm environment and are among the leading causes of bacterial diarrhea worldwide (Dai et al., 2020). Farm animals and wild birds are the primary reservoirs of *Campylobacter* spp. (Hald et al., 2015; Skarp et al., 2016), and direct contact with farm animals and poultry products are the major routes of transmission to humans (Wieczorek et al., 2020). Even though *Campylobacter* spp. rarely causes clinical disease in animals, in humans they can cause severe acute gastroenteritis (Dai et al., 2020). Campylobacteriosis, *Campylobacter*-caused enteritis, is usually self-limiting in immunocompetent individuals but it can lead to severe infections and complications in the immunocompromised (Endtz, 2020). The most common *Campylobacter* spp. associated with diarrhea in humans are *C*. *jejuni* and, to a lesser extent, *C*. *coli* (Sainato et al., 2018).

The increase of multiple and multidrug-resistant *Campylobacter* worldwide is not only attributed to the overuse of antibiotics in human medicine but also in animal farming as growth promotors and to treat and prevent infections (Blaser et al., 2021; Paintsil et al., 2021). In particular, antimicrobial-resistant *Campylobacter* poses a great risk to human health leading to treatment failures, longer hospitalization, and increased morbidity and mortality (Yang et al., 2019). Previous studies conducted in Poland (Wieczorek et al., 2020), North (Varga et al., 2019), and South America (Dias et al., 2021) demonstrated high frequencies of antibiotic-resistant *Campylobacter* in both commercial and smallholder farms. In Ghana and other parts of Africa, various studies conducted in commercial farms have also identified high frequencies of antibiotic-resistant *Campylobacter* in poultry and other livestock (Karikari et al., 2016; Dekker et al., 2019; Kunadu et al., 2020; Stringer et al., 2021; Paintsil et al., 2022).

Smallholder and commercial farming is widespread in Ghana. Poultry is the main source of meat consumed in the country (Asante-Addo and Weible, 2020). Not only poultry consumers are at risk of *Campylobacter* infections but also farmers due to their close proximity to the animals (Basler et al., 2016). Surveillance systems are largely absent in Ghana but in order to inform on circulating antibiotic-resistant *Campylobacter* and to implement effective control measures, close and continuous monitoring is required. This study investigated the prevalence and antimicrobial resistance in *C*. *jejuni* and *C*. *coli* isolated from commercial and smallholder farm animals in the Asante Akim North Municipality of Ghana.

## Materials and methods

### Study site

**Figure 1:**
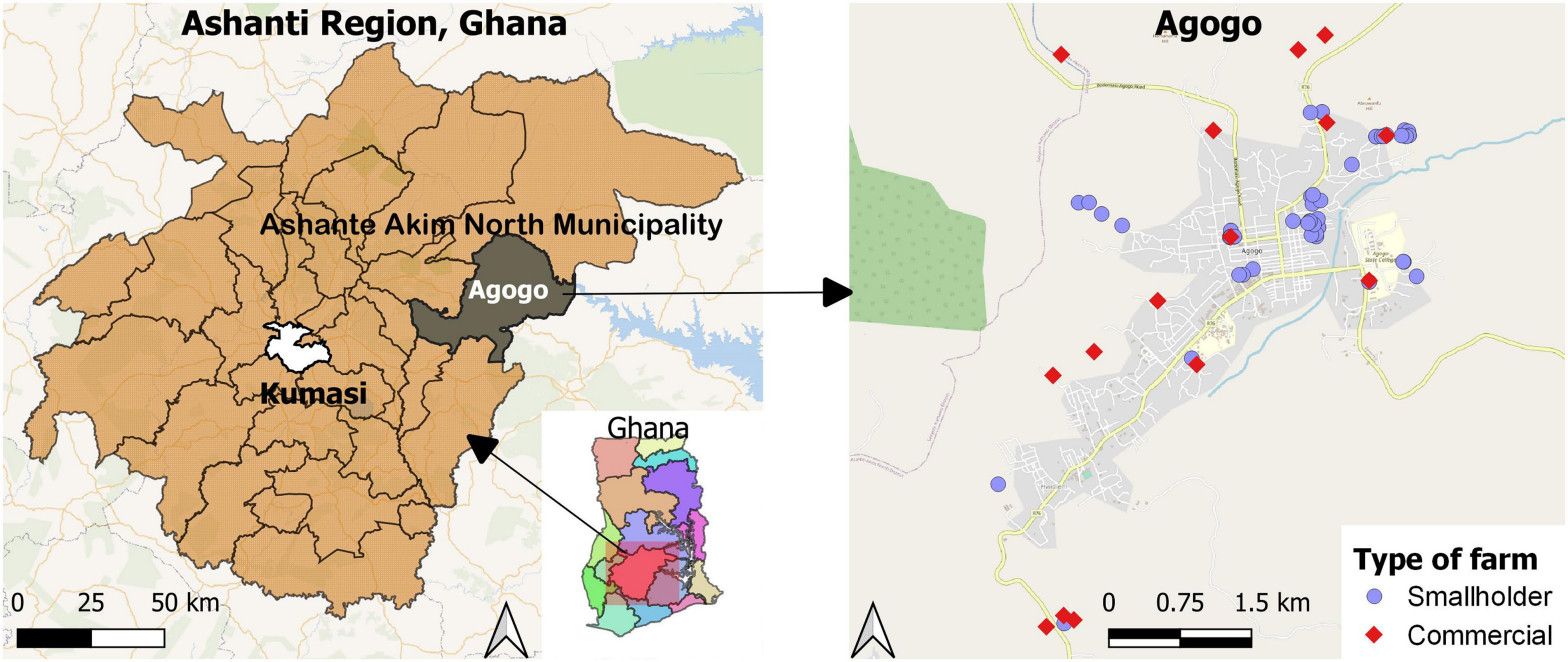
Location of commercial and smallholder farms in Agogo in the Ashanti region, Ghana.

This cross-sectional study was conducted in Agogo the capital of the Asante Akim North Municipality of Ghana (Figure 1). Asante Akim North Municipality is a rural community located in the eastern part of the Ashanti Region, with a population of 85,788 (Ghana Statistical Service, 2021). Approximately 42% of the households in the city of Agogo rear chickens, accounting for 56% of smallholder farm animals kept in the municipality (Ghana Statistical Service, 2021). The climate in the area is tropical with two main seasons. The rainy season lasts from April to October and the dry season from November to March.

### Sample collection

Sampling took place weekly between March 2019 and July 2020. Fecal samples were collected from poultry and livestock kept in commercial and smallholder farms located within the Agogo township. Poultry included chicken, turkey, duck, and quail, while livestock included cow, pig, goat, and sheep. A farm was considered commercial if it had at least 500 caged poultry and/or any quantity of caged livestock with an intensive housing system, whereas smallholder farms (small-scale agriculture) were households with free-roaming poultry (mainly of indigenous breeds) and/or livestock with shelter provided by basic or temporary roofing. A list of all commercial farms in the municipality was obtained from the municipal office of the Ministry of Food and Agriculture. All commercial farms sampled were small-scaled farms with poultry flock size ranging from 500 to 3,000 animals. Prior to sampling, the farms were visited to ascertain the number of pen houses on each farm. Commercial farms with multiple pen houses were visited more than once, however, each pen house was sampled only once during the study period. From each farm, the total samples collected were 10% of the population of farm animals kept. Sampling was done by using a sterile spatula to collect 2 g of fresh single fecal dropping. The sample was placed into a sterile plastic container without the addition of any preservatives. All samples were transported in a cool box and were further processed within 2–4 h after collection at the bacteriology laboratory of the Kumasi Center for Collaborative Research in Tropical Medicine (KCCR), Ghana.

### Identification of *Campylobacter*

Samples were transferred into an enrichment broth (Preston No. 2, Oxoid, United Kingdom) and incubated under microaerophilic conditions (CampyGen sachets in a candle jar; Oxoid) for 18–24 h at 42°C. After incubation, samples were further processed using a filter technique as described by Corry et al. (2003), and cultured on selective Karmali agar (Oxoid). Agar plates were incubated at 42°C, under microaerophilic conditions for 42–48 h. Suspicious *Campylobacter* colonies were screened for the presence of the enzyme cytochrome oxidase and Gram-staining was performed. Oxidase-positive and Gram-negative spiral-rod-shaped bacteria were stored at −80°C using the Microbank™ system. All isolates were shipped to Germany on dry ice, and species confirmation was done by Matrix-Assisted Laser Desorption Ionization Time-of-Flight Mass Spectrometry (MALDI-TOF MS) using the VITEK® MS system (bioMérieux, Marcy-l’Étoile, France).

### Antibiotic susceptibility testing

Using the disk diffusion method (Kirby Bauer), antimicrobial susceptibility testing was done on all confirmed *Campylobacter* isolates and interpreted according to the 2022 European Committee on Antimicrobial Susceptibility Testing (EUCAST) guidelines.^1^ Antibiotic disks (Oxoid) were placed on Mueller Hinton agar supplemented with 5% sheep blood inoculated with *Campylobacter*. The susceptibility plates were incubated at 42°C under microaerophilic conditions for 24 h. After 18–24 h, isolates with insufficient growth were reincubated, and the inhibition zone was read after a total of 40–48 h. Zone diameter measurements were interpreted as susceptible (S), susceptible, increased exposure (I), or resistant (R) according to EUCAST guidelines (Table 1). For antibiotics tested that did not have EUCAST clinical breakpoint for *Campylobacter*, epidemiological cut-off values (Ecoff) were established based on the frequency distribution of inhibition zone diameters (Table 1). The procedure for developing epidemiological cut-off values has been described previously (Bénéjat et al., 2018). Isolates showing resistance to at least one antimicrobial agent from each of the following antimicrobial groups: tetracyclines, macrolides, and quinolones were considered multidrug-resistant (MDR). In contrast, multiple-drug resistance was defined as resistance to three or more antimicrobials of any substance group.

**Table 1:** Breakpoints used for determination of the antimicrobial resistance of *C*. *jejuni* and *C*. *coli*.

Antibiotic (disk concentration)	Zone diameter (mm)	
	S ≥	R <
Tetracycline (30 μg)	30	30
Ciprofloxacin (5 μg)	50	26
Erythromycin (15 μg), *C*. *coli*	20	20
Erythromycin (15 μg), *C*. *jejuni*	24	24
Ampicillin (10 μg)	13^*^	7^*^
Chloramphenicol (30 μg)	18^*^	18^*^
Kanamycin (30 μg)	15^*^	7^*^
Streptomycin (25 μg)	22^*^	13^*^

* Epidemiological cut-off values (Ecoff).

### Data analysis

Descriptive analyses of *Campylobacter* detection and antibiotic susceptibility were done using absolute frequencies and their corresponding percentages. Prevalence ratios (PRs) and their respective 95% CIs were computed to show associations in bivariate analyses. Because the study is cross-sectional, PRs were used instead of odds ratios to avoid overestimation of the strength of associations (Tamhane et al., 2016). Multivariable associations with multiple drug resistance of *Campylobacter* isolates were determined using Poisson regression with robust variance (Barros and Hirakata, 2003; Greenland, 2004). The dependent variable was the presence or absence of multiple drug resistance in a *Campylobacter* isolate. Independent variables considered for regression analysis were whether the isolate was collected from a commercial or smallholder farm, rainy (April–October) or dry (November–March) season, poultry or livestock sample, and *C*. *coli* or *C*. *jejuni* species. Due to the exploratory nature of the study, no significance testing was done. All statistical analyses were performed using *R* (version 4.1.1) software (R Core Team, 2020). The *epiR* (2.0.19) package was used to calculate the PRs. The AER package (*version*: 1.2–10) was used to test for overdispersion in the Poisson model. A heatmap was created to show susceptible (S), susceptible, increased exposure (I), and resistant (R) *Campylobacter* spp. to the tested antibiotics, using the R package *gplot* (3.1.1). The *ggplot2* package (version 3.3.5) was used to plot data. QGIS software, version 3.18.3 (QGIS Development Team, Zurich, Switzerland) was used to draw a map showing the location of the commercial and smallholder farms sampled in Agogo (QGIS Development Team, 2021).

## Results

### Prevalence of *Campylobacter* spp. in commercial and smallholder poultry and livestock farms

In total, 15 commercial and 62 smallholder farms (Table 2) were sampled, of which 1,585 fecal samples were collected. These comprised 805 (50.8%) samples from commercial and 780 (49.2%) samples from smallholder farms. The majority of these fecal samples (81.8%, *n* = 1,297) were collected from poultry (i.e., chicken, turkey, duck, and quail) followed by samples from other livestock (i.e., goat, sheep, cow, and pig; 18.2%, *n* = 288). The samples produced a total of 421 (26.6%) presumptive *Campylobacter* spp., of which 75 (17.8%) were lost in the course of freeze-storage, leaving 346 (21.8%) isolates for confirmation. The confirmed prevalence of *Campylobacter* spp. found in the 1,585 collected samples was 20.3% (*n* = 322). From the 62 smallholder and 15 commercial farms, 59.7% (*n* = 37) and 86.7% (*n* = 13), respectively, were positive for *Campylobacter* spp. in at least one sample. Similar prevalence of *Campylobacte*r spp. was detected in commercial (21.0%, *n*/*N* = 169/805) and smallholder (19.6%, *n*/*N* = 153/780) farms (PR = 1.1, 95% CI: 0.9–1.3). *C*. *coli* (67.7%, *n*/*N* = 218/322) and *C*. *jejuni* (32.3%, *n*/*N* = 104/322) were the only two types of *Campylobacter* spp. isolated. But the prevalence of *C*. *coli* was 2.1 (95% CI: 1.8–2.5) times higher than *C*. *jejuni*.

**Table 2:** The prevalence of *C*. *coli* and *C*. *jejuni* in commercial and smallholder farm animals.

Sample type	*Campylobacter* spp.			
	Commercial, % (*n*/*N*)		Smallholder, % (*n*/*N*)	
	*C*. *coli*	*C*. *jejuni*	*C*. *coli*	*C*. *jejuni*
Chicken	16.0 (87/545)	9.0 (49/545)	15.2 (102/671)	5.1 (34/671)
Turkey	3.7 (1/27)	7.4 (2/27)	NA	NA
Duck	26.9 (7 /26)	0 (0/26)	NA	NA
Quail	10.7 (3 /28)	39.3 (11/28)	NA	NA
Cow	0 (0 /65)	0 (0/65)	NA	NA
Pig	11.0 (9/82)	0 (0/82)	NA	NA
Goat	0 (0/17)	0 (0/17)	9.2 (9/98)	8.2 (8/98)
Sheep	0 (0/15)	0 (0/15)	0 (0/11)	0 (0/11)
Total	13.3 (107/805)	7.7 (62/805)	14.2 (111/780)	5.4 (42/780)

n, number positive; N, total samples collected; and NA, not applicable (No sample collected).

The prevalence of *Campylobacter* spp. among poultry from commercial (25.6%, *n*/*N* = 160/626) and smallholder (20.3%, *n*/*N* = 136/671) farms was similar (PR = 1.3, 95% CI: 1.0–1.5). However, more *Campylobacter* spp. was isolated from livestock in smallholder farms (15.6%, *n*/*N* = 17/109) than commercial farms (5.0%, *n*/*N* = 9/179; PR = 3.1, 95% CI: 1.4–6.7). Quails from commercial farms showed the highest prevalence of *C*. *jejuni* (39.3%, *n*/*N* = 11/28), while ducks from commercial farms accounted for the highest *C*. *coli* prevalence (26.9%, *n*/*N* = 7/26; Table 2). In chicken, similar numbers of *Campylobacter* spp. were isolated from commercial (25.0%, *n*/*N* = 136/545) and smallholder (20.3%, *n*/*N* = 136/671) farms (PR = 1.2, 95% CI: 1.0–1.5). Table 2 gives further details of the frequency of *C*. *coli* and *C*. *jejuni* detected from the 1,585 poultry and livestock fecal samples collected from commercial and smallholder farm animals. No *Campylobacter* spp. was isolated from sheep or commercially reared goats and cows.

### Seasonal prevalence of *Campylobacter*

**Figure 2:**
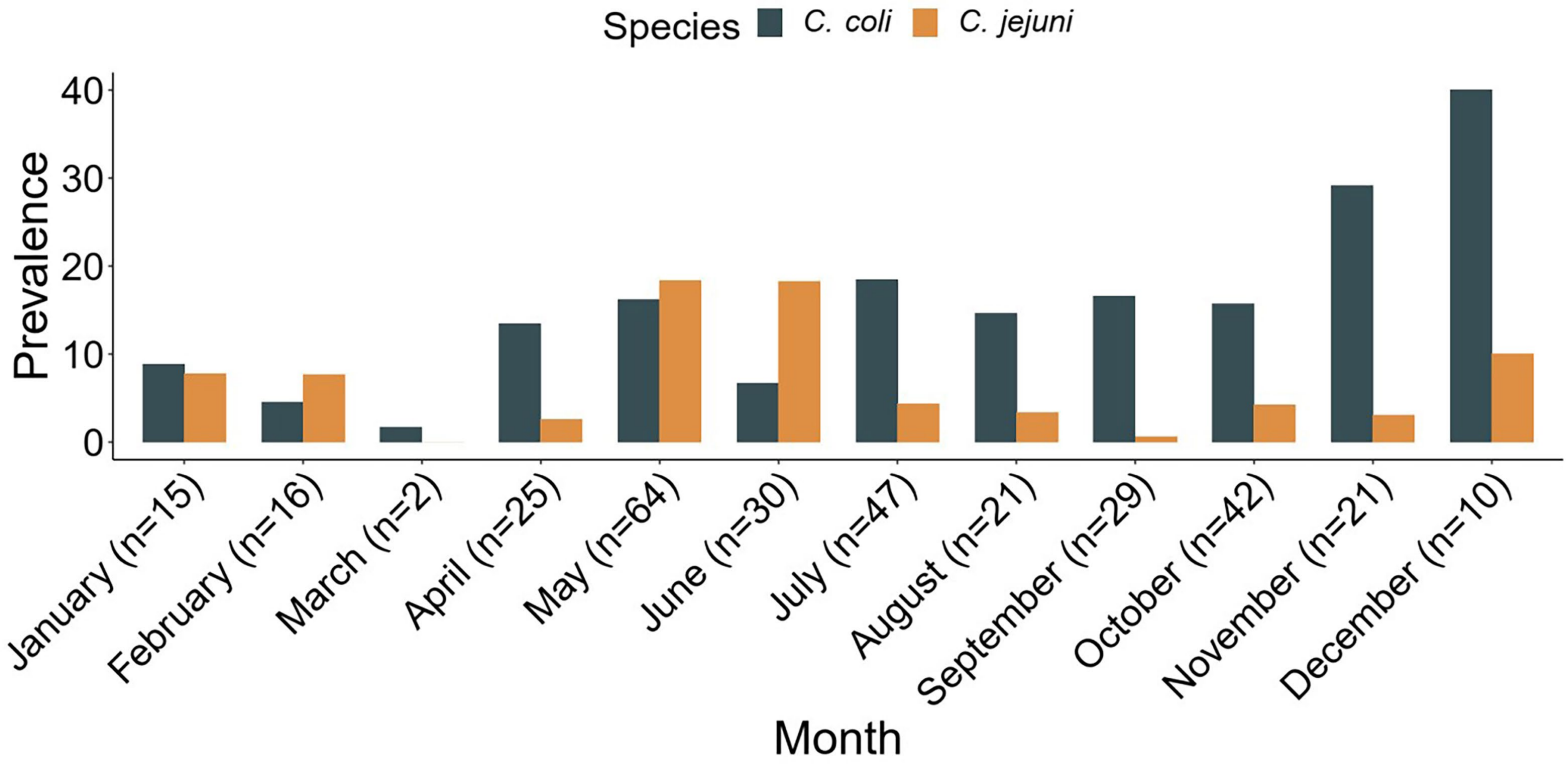
Seasonal *Campylobacter jejuni* and *Campylobacter coli* prevalence (%). The total number of *Campylobacter* spp. isolated within each month is represented by *n*.

The seasonal prevalence by month of *Campylobacter*, including *C*. *coli* and *C*. *jejuni*, is shown in Figure 2. The result shows that *Campylobacter* spp. were isolated throughout the year with a prevalence ranging from 1.7% (*n*/*N* = 2/120) in March up to 50.0% (*n*/*N* = 10/20) in December, although only 20 samples were tested in the latter month. The months of May (34.6%, *n*/*N* = 64/185), November (32.3%, *n*/*N* = 21/65), and June (25.0%, *n*/*N* = 30/120) recorded the highest *Campylobacter* prevalence, while January to April had the lowest prevalence (range = 1.7–16.7%). *Campylobacter* isolation rate in the rainy season (April to October) was 22.2% (*n*/*N* = 258/1,160), which was higher than the 15.1% (*n*/*N* = 64/425) recorded in the dry season (November–March; PR = 1.5, CI: 1.2–1.9).

### Antibiotic resistance of *Campylobacter coli* and *Campylobacter jejuni*

Ecoff values were derived for Ampicillin (10 μg), Chloramphenicol (30 μg), Kanamycin (30 μg), and Streptomycin (25 μg) (Supplementary File 1). Apart from chloramphenicol, for which no antibiotic resistance was detected, overall higher antibiotic resistance was observed for both *C*. *coli* and *C*. *jejuni* isolated from commercial farms compared to smallholder farms (Table 3). *Campylobacter jejuni* isolated from smallholder farms showed low resistance to ciprofloxacin (16.7%, *n*/*N* = 7/42), streptomycin (14.8%, *n*/*N* = 6/42), and tetracycline (11.9%, *n*/*N* = 5/42; Table 3). Compared to smallholder farms, animals from commercial farms were at least 50% more likely to be resistant to kanamycin (PR = 19.7; 95% CI: 2.7–144.7), erythromycin (PR = 4.2; 95% CI: 1.4–12.0), tetracycline (PR = 3.7; 95% CI: 2.4–5.5), and ciprofloxacin (PR = 1.7; 95% CI: 1.3–2.1). Compared *to C*. *jejuni*, *C*. *coli i*solates from both commercial and smallholder farms had higher resistance rates to kanamycin (PR = 9.5, 95% CI: 1.3–70.1) and ciprofloxacin (PR = 1.4, 95% CI: 1.1–1.8; Table 3).

**Table 3:** Antibiotic resistance of *C*. *coli* and *C*. *jejuni* isolated from commercial and smallholder farm animals.

Antibiotic	Resistance, % (n)					
	Commercial (N)		Smallholder (N)		Total (N)	
	*C*. *coli* (107)	*C*. *jejuni* (62)	*C*. *coli* (111)	*C*. *jejuni* (42)	*C*. *coli* (218)	*C*. *jejuni* (104)
Ciprofloxacin	73.8 (79)	58.1 (36)	44.1 (49)	16.7 (7)	58.7 (128)	41.3 (43)
Tetracycline	69.2 (74)	80.6 (50)	18.9 (21)	11.9 (5)	43.6 (95)	52.9 (55)
Streptomycin	23.4 (25)	21.0 (13)	17.1 (19)	14.8 (6)	20.2 (44)	18.3 (19)
Ampicillin	24.3 (26)	30.6 (19)	0 (0)	4.8 (2)	11.9 (26)	20.2 (21)
Kanamycin	17.8 (19)	0 (1)	0 (1)	0 (0)	9.2 (20)	0 (1)
Erythromycin	15.0 (16)	0 (0)	3.6 (4)	0 (0)	9.2 (20)	0 (0)
Chloramphenicol	0 (0)	0 (0)	0 (0)	0 (0)	0 (0)	0 (0)

n, Number of resistant isolates; N, total isolates.

**Figure 3:**
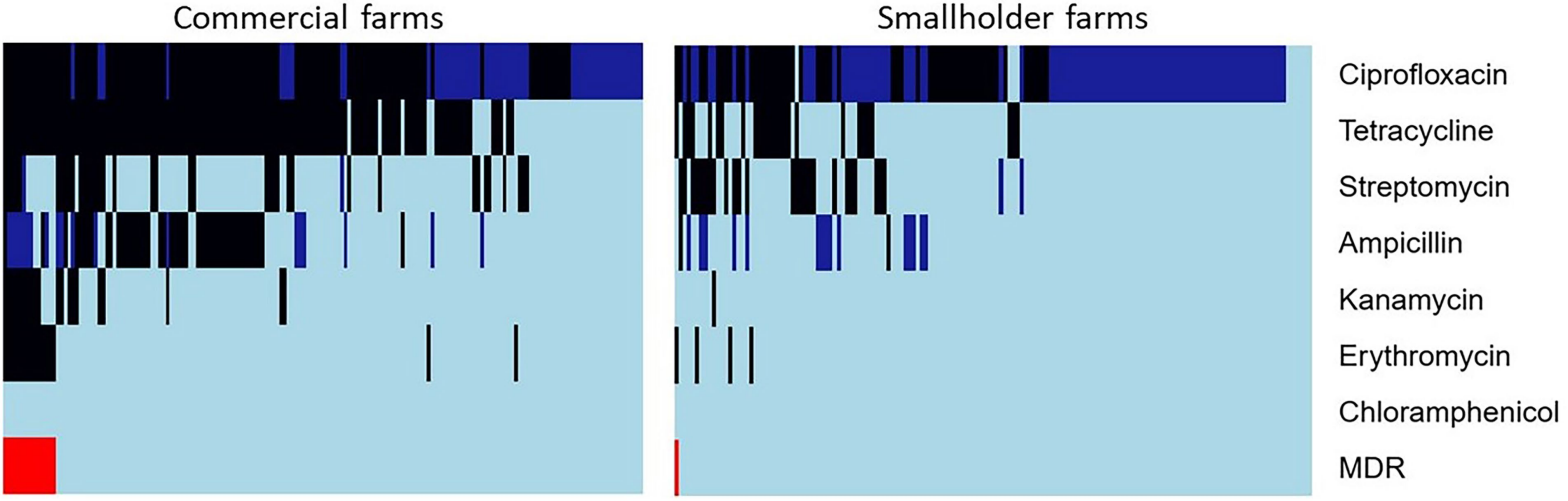
Antibiotic susceptibility profiles of *Campylobacter* isolates (columns) from commercial and smallholder farms. The color of each cell represents susceptible (S, light blue), susceptible, increased exposure (I, deep blue), resistant (R, black), and multidrug resistance (MDR, red).

The heatmap in Figure 3 shows *Campylobacter* spp. either susceptible (S), susceptible at increased exposure (I), or resistant (R) to the tested antibiotics. Only six isolates (1.7%, *n*/*N* = 6/322), all from smallholder farms, were susceptible to all seven antibiotics tested. The majority of ampicillin (95.7%, *n*/*N* = 45/47), kanamycin (95.2%, *n*/*N* = 20/21), tetracycline (82.7%, *n*/*N* = 124/150), erythromycin (80%, *n*/*N* = 16/20), and ciprofloxacin (67.3%, *n*/*N* = 115/171) resistance were observed among isolates from commercial farms. Almost half (43.8%, *n*/*N* = 141/322) of the *Campylobacter* isolates from both commercial and smallholder farms showed susceptibility at increased exposure (I) to ciprofloxacin. Interestingly, no *Campylobacter* from commercial farms was susceptible (S, standard dosing regimen) to ciprofloxacin and only 6.5% (*n*/*N* = 10/322) of isolates from smallholder farms were susceptible to this antibiotic.

### Poisson regression analysis of factors associated with drug-resistant *Campylobacter* spp.

Multiple drug resistance (i.e., resistance to three or more antibiotics) was observed for 23.3% (*n*/*N* = 75/322) of the *Campylobacter* spp. isolated in this study. A vast majority (97.3%, *n*/*N* = 73/75) of the recorded multiple drug resistance occurred in *Campylobacter* spp. isolated from commercial farm animals. Table 4 summarizes factors associated with multiple drug resistance in *Campylobacter* from commercial and smallholder farms. Isolates recovered from commercial farms and isolates collected during the rainy season were more likely to show multiple drug resistance. However, the species of the isolates and the type of animal it was recovered from did not show strong associations with multiple drug resistance.

**Table 4:** Associations with the probability of having multidrug-resistant *Campylobacter* spp.

Variable	Crude Ratio PR (95% CI)	Adjusted Ratio PR (95% CI)
Commercial vs. smallholder farm	14.9 (5.6–40.0)	14.5 (5.5–37.9)
Rainy vs. dry season	5.5 (1.8–17.0)	4.9 (1.6–14.5)
Poultry vs. livestock	2.9 (0.7–11.5)	1.8 (0.5–6.8)
*C*. *coli* vs. *C*. *jejuni*	1.2 (0.7–1.9)	1.4 (1.0–2.2)

PR, Prevalence Ratio.

Multidrug resistance (i.e., resistance to the antibiotics typically used for the treatment of campylobacteriosis: erythromycin, tetracycline, and ciprofloxacin) was observed for 4.7% (*n*/*N* = 15/322) of the *Campylobacter* spp. isolated in this study (Figure 3). All the MDR *Campylobacter* were *C*. *jejuni* isolated during the rainy season. A vast majority (93.3%, *n*/*N* = 14/15) of the MDR occurred in commercial farms hence MDR *Campylobacter* spp. was 12.7 (95% CI: 1.7–95.3) more likely to be isolated from commercial farms than smallholder farms.

## Discussion

In the present study, results on the prevalence of antibiotic-resistant *Campylobacter* from commercial and smallholder farm animals in Agogo in the Ashanti region of Ghana were described. Overall, contamination *with Campylobacter* spp. in both commercial and smallholder farms was significantly high. These findings are in line with established knowledge, that poultry and livestock are major reservoirs of *Campylobacter* spp., worldwide (Sibanda et al., 2018; Plishka et al., 2021). Similar to this study, very high levels of *Campylobacter* prevalence have been detected at commercial farm levels (93%) in the United States (Zhang and Sahin, 2020); these high prevalence levels are likely due to intensive husbandry conditions (Gilbert et al., 2021). However, for smallholder farms, the frequency observed in our study was slightly higher than the 42.4% reported by a study conducted on similar types of farms in peri-urban Addis Ababa, Ethiopia (Chala et al., 2021). Higher *Campylobacter* frequencies are generally expected in poultry, so the differences in frequencies might be because the latter study was conducted on farms that kept only livestock.

The prevalence of *Campylobacter* spp. isolated from poultry from commercial and smallholder farms studied in Agogo were almost equally high. However, the *Campylobacter* prevalence in poultry from commercial farms was much lower than what was reported earlier in Burkina Faso (68%; Kagambèga et al., 2018), Poland (53.4%; Wieczorek et al., 2020), and China (56.1%; Han et al., 2016). The seemingly lower prevalence reported here might be due to differences in study methodologies, husbandry conditions, and study populations. Nonetheless, the *Campylobacter* prevalence in poultry from commercial farms identified in the present study was higher than 18% (Kunadu et al., 2020) and comparable to the 22.5% (Karikari et al., 2016) observed in similar studies conducted in Ghana. In smallholder poultry farms in Agogo, the *Campylobacter* prevalence observed in our study is consistent with the 17.7% reported by a previous study conducted in free-range broiler breeder flocks in the United Kingdom (Colles et al., 2015). Also, we found relatively high frequencies of *Campylobacter* in pigs from commercial farms and goats from smallholder farms. Similar to the current findings, a high prevalence of *Campylobacter* has been recorded in pigs and goats from Sub-Saharan Africa (Gahamanyi et al., 2020).

Our study isolated the species: *C*. *coli* and *C*. *jejuni* only. Apart from quails which recorded more *C*. *jejuni* isolation than *C*. *coli*, the overall prevalence of *C*. *coli* was twice as high than what was found for *C*. *jejuni*. There is no consensus on which of the two *Campylobacter* species, *C*. *coli* and *C*. *jejuni*, is dominant in poultry and livestock. In agreement with our findings, recent studies from Africa, Asia, and Europe have reported significantly more *C*. *coli* in chicken (Torralbo et al., 2015; Wieczorek et al., 2020), ducks (Uddin et al., 2021), poultry meat (Dekker et al., 2019), and pigs (Padungtod and Kaneene, 2005; Wieczorek et al., 2021). On the contrary, several studies have found almost exclusively *C*. *jejuni* in chicken (Guyard-Nicodème et al., 2015; Karikari et al., 2016), poultry meat (Szosland-Fałtyn et al., 2018), quails (Cox et al., 2018), and wild birds (Hald et al., 2015). One possible *contributing reason* why *C*. *coli* and *C*. *jejuni* are the most prevalent and pathogenic *Campylobacter species* is because they have longer viability in the environment compared to other species, hence increasing their chance of survival and recovery (Stringer et al., 2021).

In the present study, the frequency of *Campylobacter* was higher in the rainy season (22.2%) than in the dry season (15.1%). Seasonal variations in the prevalence of *Campylobacter and other* bacteria that cause diarrhea have been demonstrated by several researchers. In temperate regions, *Campylobacter* prevalence is typically highest during the summer months of the year with higher temperatures (Djennad et al., 2019; Wieczorek et al., 2020). The association between seasonal variations and *Campylobacter* prevalence appears to be indirect (Djennad et al., 2019). The higher *Campylobacte*r prevalence recorded in the rainy season by the current study is in line with studies from other parts of Africa (Mandomando et al., 2007; Adam et al., 2018).

In the present study, the derived Ecoff values were comparable to cut-off values reported by Frediani-Wolf and Stephan (2003). Almost all isolates from our study were resistant to at least one antimicrobial drug and MDR was detected for 4.7% of the isolates. The level of resistance observed is consistent with reports from Benin (Kouglenou et al., 2020) and Germany (El-Adawy et al., 2015). Resistance to chloramphenicol was not detected in any of the *Campylobacter* isolates. A study conducted in Ethiopia, in households that owned livestock, reported an increased rate of chloramphenicol (19.4%) resistance (Stringer et al., 2021). Differences might be attributed to the fact that chloramphenicol is not typically used in animal husbandry in our study area (Paintsil et al., 2021).

Higher overall frequencies of antibiotic resistance were seen for *C*. *coli* and *C*. *jejuni* from commercial farms. An earlier study performed in the same study area found that 97% of commercial farms used antibiotics as compared to 47% of smallholder farms (Paintsil et al., 2021). Therefore, higher antibiotic use in commercial farming may explain these findings. In the current study, the majority of *C*. *coli* compared to *C*. *jejuni* were resistant to ciprofloxacin and ampicillin. Similar to our findings, several researchers have observed that *C*. *coli* exhibited higher resistance than *C*. *jejuni* (Ocejo et al., 2019; Wieczorek et al., 2020). One probable hypothesis for this observation could be a higher capacity of *C*. *coli* to acquire resistant genes by horizontal gene transfer (Golz and Stingl, 2021). Nonetheless, in the absence of fluoroquinolone use, *C*. *coli* isolates displayed lower resistance to tested antibiotics than *C*. *jejuni* (Abraham et al., 2020). The high frequency of ciprofloxacin resistance observed in this study is worrisome because ciprofloxacin is one of the most important antibiotics used in the treatment of campylobacteriosis, also in Ghana (WHO, 2019).

There are few limitations to the present study that need to be considered when interpreting our findings. The farms selected and sample collection was restricted to the Ashanti Akim District of Ghana, hence, the data observed might not be representative of the whole of Ghana. This is because *Campylobacter* frequencies, antibiotic resistance profiles as well as species distribution might be subject to geographic variations. In total, almost 20% of presumptively isolated *Campylobacter* were lost in the course of freeze-storage; hence, the use of a different identification method, such as direct PCR, could have affected the observed *Campylobacter* prevalence. Also, there is potential clustering of resistance in the *Campylobacter* isolates due to easy access to similar types of antibiotics in the study area. Hence the Ecoff values established may have locally constrained lower and upper bounds as well as variance which may not be globally applicable.

## Conclusion

While *Campylobacter* frequencies from commercial and smallholder farms were similarly high, antibiotic resistance was considerably lower in smallholder farms. Isolates recovered from commercial farms or isolated during the rainy season were more likely to be MDR. The occurrence of high antibiotic-resistant *Campylobacter* in commercial farm animals could lead to the emergence and distribution of drug-resistant *Campylobacter* in humans who consume or come into contact with the animals. Hence, a better understanding of the reasons for the observed differences in *Campylobacter* prevalence and MDR in the two farm types would make it possible to formulate clear guidelines aimed at decreasing prevalence and resistance for safe animal husbandry. We further recommend that farm attendants should be trained on personnel hygiene, farm biosecurity, appropriate use of antimicrobials, and the need for AMR surveillance monitoring systems in farm animal production.

## Data availability statement

The raw data supporting the conclusions of this article will be made available by the authors, without undue reservation.

## Ethics statement

The animal study was reviewed and approved by Committee on Human Research Publication and Ethics. Written informed consent was obtained from the owners for the participation of their animals in this study.

## Author contributions

DD, LAO, KO-D, ROP, JMa, AEZ, and RK: conceptualization, resources, supervision, and review and editing. EKP: original draft preparation. EKP and RK: formal analysis. EKP, CWA, JMb, AJ, and ML: methodology, data curation, investigation, and review and editing. All authors contributed to the article and approved the submitted version.

## Funding

This work was funded by the German Research Foundation (DFG; project number 380545990).

## Conflict of interest

The authors declare that the research was conducted in the absence of any commercial or financial relationships that could be construed as a potential conflict of interest.

## Publisher’s note

All claims expressed in this article are solely those of the authors and do not necessarily represent those of their affiliated organizations, or those of the publisher, the editors and the reviewers. Any product that may be evaluated in this article, or claim that may be made by its manufacturer, is not guaranteed or endorsed by the publisher.

## Footnotes

^1^ http://www.eucast.org
